# The keratinolytic bacteria *Bacillus cytotoxicus* as a source of novel proteases and feather protein hydrolysates with antioxidant activities

**DOI:** 10.1186/s43141-021-00207-1

**Published:** 2021-07-22

**Authors:** Ivana Cavello, Brenda Bezus, Sebastián Cavalitto

**Affiliations:** Centro de Investigación y Desarrollo en Fermentaciones Industriales. Facultad de Ciencias Exactas, Universidad Nacional de la Plata (CINDEFI, CCT La Plata-CONICET, UNLP), Calle 47 y 115, (B1900ASH), La Plata, Argentina

**Keywords:** Antioxidant activity, *Bacillus cytotoxicus*, Feather protein hydrolysate, Proteases, Thermotolerant bacteria

## Abstract

**Background:**

Argentina’s geothermal areas are niches of a rich microbial diversity. In 2020, species of *Bacillus cytotoxicus* were isolated for the first time from these types of pristine natural areas. *Bacillus cytotoxicus* strains demonstrated the capability to grow and degrade chicken feathers with the concomitant production of proteases with keratinolytic activity, enzymes that have multitude of industrial applications. The aim of this research was to study the production of the proteolytic enzymes and its characterization. Also, feather protein hydrolysates produced during fermentation were characterized.

**Results:**

Among the thermotolerant strains isolated from the Domuyo geothermal area (Neuquén province, Argentina), *Bacillus cytotoxicus* LT-1 and Oll-15 were selected and put through submerged cultures using feather wastes as sole carbon, nitrogen, and energy source in order to obtain proteolytic enzymes and protein hydrolysates. Complete degradation of feathers was achieved after 48 h.

Zymograms demonstrated the presence of several proteolytic enzymes with an estimated molecular weight between 50 and > 120 kDa. Optimum pH and temperatures of *Bacillus cytotoxicus* LT-1 crude extract were 7.0 and 40 °C, meanwhile for Oll-15 were 7.0 and 50 °C. Crude extracts were inhibited by EDTA and 1,10 phenanthroline indicating the presence of metalloproteases.

Feather protein hydrolysates showed an interesting antioxidant potential measured through radical-scavenging and Fe^3+^-reducing activities.

**Conclusion:**

This work represents an initial approach on the study of the biotechnological potential of proteases produced by *Bacillus cytotoxicus*. The results demonstrated the importance of continuous search for new biocatalysts with new characteristics and enzymes to be able to cope with the demands in the market.

## Background

Food industry, especially poultry industry, generates large quantities of feathers (organic wastes) which are mainly composed by keratin (almost 90%). The growing intensity of this industrial activity leads to the accumulation of this kind of hard-to-degrade feather waste and causes a complete need for its disposal. Many countries have adopted the burning strategy, but it is highly polluting to the atmosphere. Alternatively, the production of feather meal by chemical treatment at high pressure can partially hydrolyze this recalcitrant protein, increasing the digestibility but reducing the amount of certain thermolabile amino acids, limiting its use as a livestock feed additive [1].

Microbial degradation has become the most suitable form of hydrolyzing this protein, offering an eco-friendly method, which is in concordance with the green practices that are necessary to adapt to protect the environment [1]. The bioconversion of feathers by keratinolytic microorganisms results in the production of enzymes such as keratinases and proteases, microbial biomass, and protein hydrolysates (with amino acids and peptides with bioactive properties). Keratinases and proteases are very useful for many industrial applications, especially on detergent industry, leather and textile industry, prion decontamination, and certain medical and cosmetic applications [1, 2].

The growing interest on the revalorization of feather waste to produce enzymes and protein hydrolysates reveals the need for further research, focusing on the search of enzyme systems to convert organic wastes into bioactive peptides which could be used for the formulation of novel bioproducts [3].

Although many proteases have been described from thermophilic and hyperthermophilic bacteria, only few thermophilic/thermotolerant bacteria are known to produce proteases with keratinolytic activity [4–9].

This study aimed to describe the production and characterization of the proteolytic extracts and the in vitro bioactive potentials of the culture supernatants produced by thermotolerant keratinolytic species of *Bacillus cytotoxicus* during submerged fermentation, with whole feathers as only carbon and nitrogen sources. As far as we know, this is the first approach to characterize the enzyme cocktail that these microorganisms produce.

## Methods

### Microorganisms

The strains *Bacillus cytotoxicus* LT-1 and *B. cytotoxicus* Oll-15, previously isolated from samples collected from the hot springs of Los Tachos and Las Olletas in the Domuyo geothermal area, were used in this study. They are deposited on the Microbiological Culture Collection of CINDEFI-CONICET Institute, La Plata, Buenos Aires, Argentina, and maintained on nutrient agar slants or glycerol stock (20% w/v) at 4 and – 80 °C.

The strains were previously identified as *B. cytotoxicus* not only by their 16S rRNA gene sequences but also by the amplification of *cytK-1* gene [10, 11].

### Growth conditions and protease production

Submerged fermentations were performed using 250-ml Erlenmeyer flasks with 50 ml of minimal mineral media (MMM) supplemented with 10 g·l^-1^ of whole chicken feather. MMM was composed of 2.486 g·l^-1^ K_2_HPO_4_, 0.496 g·l^-1^ NaH_2_PO_4_, 0.01 g·l^-1^ MgCl_2_, 0.016 g·l^-1^ FeCl_3_, 0.1 mg·l^-1^ CaCl_2_, and 0.013 g·l^-1^ ZnCl_2_, with pH 6.0 [12]. Chicken feathers were obtained from a local slaughterhouse and were washed with 0.1% (w/v) sodium dodecyl sulfate (SDS) and with 1:1 (v/v) methanol and water with shaking for 18 h before use.

MMM supplemented with whole chicken feathers were inoculated with 1 ml of a 24-h nutrient broth culture (OD_i 600_ 0.5) of each bacterial strain and incubated at 50 °C, 150 rpm for 2 days. Samples were withdrawn in sterile conditions periodically. Biomass and other residues were separated by centrifugation at 10,000 × *g* for 10 min at 4 °C, and supernatants were used as crude extracts for the subsequent analysis.

### Production of feather protein hydrolysates (FPHs)

FPHs were obtained inoculating the bacterial strains on MMM supplemented with 10 g·l^-1^ of whole chicken feathers and incubating them at 50 °C, 150 rpm until feather degradation was completed (2 days). Then, cultures were centrifuged at 6000 *× g* for 15 min, filtered through a 0.54-μm cellulose acetate filter, and the supernatants were preserved at – 20 °C until the performance of antioxidant assays.

### Enzyme activity

Proteolytic activity was determined using azocasein (Sigma Co., USA) as substrate following the protocol described by Cavello et al. [12]. Briefly, 100 μl of suitable dilution of each enzyme preparation were added to 250 μl of the substrate (10 g·l^-1^, Tris-HCl buffer 20 mM; pH 7.0). The admixture was incubated for 1 h at 50 °C, and after that, the reaction was stopped by the addition of 1 ml of trichloroacetic acid (TCA; 10%, w/v). After centrifugation (10,000 *× g* for 15 min, 4 °C), equal volumes of the supernatant and NaOH (1.0 M) were mixed. The absorbance at 440 nm was measured. Blanks were performed for each reaction. One unit (U) of protease activity was defined as the amount of enzyme that produced an increase in 0.01 absorbance units under the experimental assay conditions described [12].

### Protein determination

The protein concentration of the samples were performed by the Bradford method [13], using bovine serum albumin (SIGMA) as standard.

### Effect of pH on the enzyme activity

Optimum pH was determined by incubating each enzymatic extract with azocasein as substrate at different pH levels (6.0–12.0, MES-Tris-Glycine buffers 20 mM each). After 1 h of incubation at 50 °C, residual activity was performed, and percentage relative activity was calculated considering 100% of the activity was displayed with the optimum pH.

### Effect of temperature on the activity and stability

Effect of temperature on enzyme activity was studied by incubating the reaction admixture (enzyme + substrate) at different temperatures (20–80 °C). Enzyme activity has been expressed as percentage relative activity.

Activation energies (Ea) of the enzymes were calculated by plotting the residual activity after the incubation at different temperatures according to the linearized Arrhenius equation (1):

1 $$ \ln K=\frac{\mathrm{Ea}}{R}\ \frac{1}{T}+\ln A, $$

where *K* is the enzymatic activity rate at the correspondent temperature (*T*, in Kelvins), Ea is the activation energy, *R* is the gas constant (8.314 J·K^-1^·mol^-1^), and *A* is a constant.

Thermal stability was determined by incubating the extracts at different temperatures between 30 and 50 °C. Aliquots were withdrawn at different times, and residual activities were measured considering 100% of activity was displayed by the non-treated enzyme.

### Effect of protease inhibitors, metal ions, and organic solvents on protease activity

The nature of the proteases present on the enzymatic extracts was studied by evaluating the effect of different protease inhibitors: phenylmethylsulfonyl fluoride (PMSF, 1 mM), ethylenediaminetetraacetic acid (EDTA, 1 mM), 1,10-phenanthroline (1 mM), iodoacetamide (1 mM), and pepstatin A (5 μM). Extracts were incubated with each inhibitor separately for 1 h at 20 °C, and after that, standard assay of proteolytic activity was performed. The activity obtained by the enzyme without any inhibitor-incubation was taken as 100% of activity.

The effect of different metal ions (MgCl_2_, CoCl_2_, CaCl_2_, MnCl_2_, ZnCl_2_, NaCl, KCl, and HgCl_2,_ 1 mM) on the enzymatic activity was evaluated incubating each extract for 60 min at 20 °C. After that, the residual activity was determined, and the activity of the enzyme without metal ion was considered as 100%.

Concerning to the effect of some organic solvents on enzyme activity, methanol, ethanol, and isopropanol (1% v/v) were incubated with extracts for an hour at 20 °C. After that, the residual enzymatic activity was determined and expressed as a percentage of the non-incubated enzyme.

### Stability and compatibility with laundry detergents

Stability and compatibility of *B. cytotoxicus* extracts with solid (Drive, Ariel, Ace or Skip, 7 mg·l^-1^) and liquid (Ace, Ala, Ariel, or Skip, 1% v/v) laundry detergents were studied, preincubating each laundry detergent with each enzymatic extract for 1 h at room temperature. After incubation, the remaining activity was determined under standard assay conditions, and residual activity was calculated considering 100% of the activity was displayed by the enzyme incubated without the addition of any detergent. Before the assay, endogenous enzymes present on the commercial detergents were inactivated by a heat treatment of 65 °C for 1 h.

### Sodium dodecyl sulfate polyacrylamide gel electrophoresis (SDS-PAGE) and zymogram

SDS-PAGE coupled to a zymogram was performed as described before by García-Carreño et al. [14], with some modifications. A 5% w/v stacking gel and 12% w/v separating gel were prepared as described by Laemmli [15]. To carry out the zymogram, after electrophoresis, SDS was removed from the gel flooding it with a solution of Triton X-100 (2.5% w/v Tris-HCl 20 mM pH 7.0) under agitation for 1 h. Then, the gel was washed three times with Tris-HCl 20 mM and incubated with casein 10.00 g·l^-1^ for 1 h at 50 °C. After incubation, the gel was stained with Coomassie Brilliant Blue G-250 following the Coomassie Colloidal method [16]. The presence of protease activity was evidenced by a clear zone in a blue background. For the SDS-PAGE, a broad range molecular weight marker was used (Thermo Fisher Scientific, #26612).

### Antioxidant activity

#### Reducing power assay

To determine the ability of the feather protein hydrolysates to reduce Fe (III), 100 μl of sample were incubated with 250 μl of phosphate buffer (0.2 M, pH 6.6) and 250 μl of K_3_Fe(CN)_6_3H_2_O 10.00 g·l^-1^ at 50 °C for 30 min. After that, 250 μl of 10% w/v TCA were added. The admixture was centrifuged at 16,000 *× g* for 10 min, and 250 μl of the supernatant was mixed with 250 μl of water and 50 μl of FeCl_3_ (0.1% w/v). After 10 min, absorbance at 700 nm was measured, and reducing power was identified in samples that produced an increase of the absorbance. Results were expressed as absorbance at 700 nm. As positive control, the reducing power of ascorbic acid was also determined (0–3.0 g·l^-1^). Blanks were performed using not inoculated media instead of FPH [17].

#### ABTS radical scavenging activity

ABTS (2,20-azino-bis-(3-ethylbenzothiazoline)-6-sulfonic acid) radical cation (ABTS^*+^) was produced by reacting ABTS stock solution (3.60 g·l^-1^) with potassium persulfate (0.66 g·l^-1^) allowing the mixture to stand in the dark for at least 12 h at 20 °C before use [18]. For the assay, a fresh solution of ABTS^*+^ was prepared by dilution with 5 mM phosphate-buffered saline (PBS, pH 7.4) to reach an absorbance of 0.7 ± 0.05 at 734 nm. Sample (10 μl) was mixed with ABTS^*+^ solution (1 ml), and the absorbance (734 nm) was taken after 4 min of reaction. A calibration curve with Trolox (6-Hydroxy-2,5,7,8-tetramethylchromane-2-carboxylic acid) was performed (0–5.0 mM). Results were expressed as the milliequivalents (mEq) of Trolox displayed per gram of feather protein hydrolysate.

The radical scavenging activity of the sample was calculated as shown in equation 2:

2 $$ \mathrm{ABTS}\ \mathrm{radical}\ \mathrm{scavenging}\ \mathrm{activity}\ \left(\%\right)=\frac{A_B-{A}_S}{A_B}\times 100 $$

where *A*_*B*_ is the absorbance at 734 nm of the blank, and *A*_*S*_ is the absorbance at 734 nm of the sample reaction. Blank reactions were performed using non-inoculated media instead of FPH.

Half maximal effective concentration (EC_50_) values were calculated from the percentage radical scavenging against FPH concentration plots and represent the concentration of FPH resulting in 50% radical scavenging.

### Nucleotide sequence accession number

Sequences of 16S rRNA gene of strains LT-1 and Oll-15 are deposited in the GenBank database under accession numbers KR559937 and KR559942, respectively [11].

### Statistical analysis

Results were expressed as an average of three independent experiments with the correspondent standard deviation. *P* values less or equal to 0.05 were considered as statistically significant (LSD test of ANOVA).

## Results

### Microorganisms

In the southwest of Argentina, there are two volcanic geothermal areas: Copahue and Domuyo. Prokaryotic biodiversity and the biotechnological applications of some of the species isolated in Copahue have been quite studied; meanwhile, the Domuyo geothermal area remains almost unexplored in these aspects [19–23].

It is in the Domuyo geothermal area that several strains of *Bacillus cytotoxicus* were isolated and characterized*,* a novel keratinolytic bacteria isolated for the first time from pristine environments. Among the collection of *B. cytotoxicus* strains, *B. cytotoxicus* LT-1 and *B. cytotoxicus* Oll-15 were selected due to its capability to grow and degrade whole chicken feathers on a minimal mineral media after only 24–48 h of cultivation at 50 °C [11].

### Protease production

**Fig. 1:**
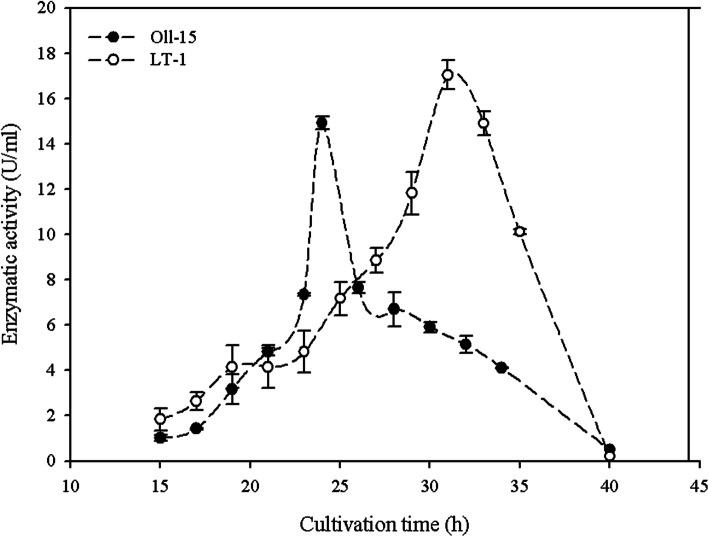
Keratinolytic protease production profiles of *B. citotoxycus* strains. Strains were incubated in MMM supplemented with 10 g·l^-1^ of whole feather at 50 °C for 48 h

Time course production of proteases is presented in Fig. 1. *Bacillus cytotoxicus* Oll-15 under the described fermentation conditions reached a maximum enzyme production of 14.4 ± 0.2 U·ml^-1^ in 24 h of incubation, while strain LT-1 reached a maximum of 16.6 ± 0.4 U·ml^-1^ after 33 h of cultivation.

### Effect of pH on the activity

**Fig. 2:**
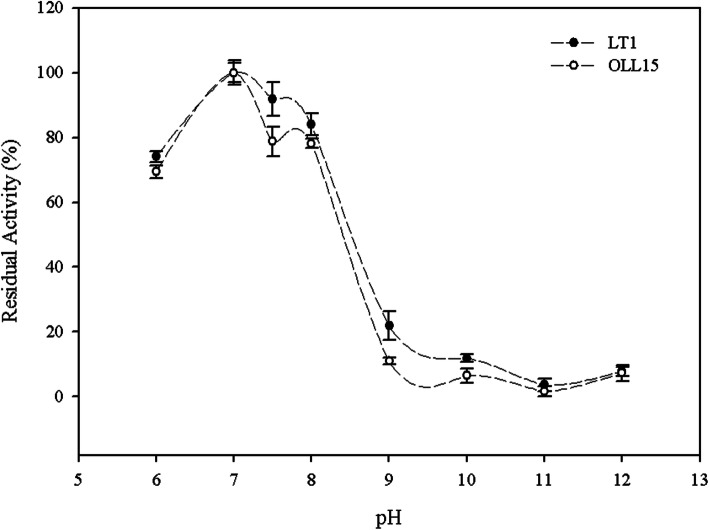
Effect of pH on the activity of proteolytic extracts obtained with *Bacillus cytotoxicus* LT-1 and *Bacillus cytotoxicus* Oll-15. Each determination was performed in triplicates, and values represent mean ± SD

The study on the effect of pH on the enzymatic activity demonstrated that both proteolytic extracts displayed optimum activity at neutral pH (pH 7.0, Fig. 2). It can be seen as a considerable residual activity in the range of pH 6.0–8.0 (higher than 69% for Oll-15 and 92% for LT-1, respectively). A decrease in residual activity at pH higher than 9.0 was observed for both enzymatic extracts.

### Effect of temperature on the activity and stability

**Fig. 3:**
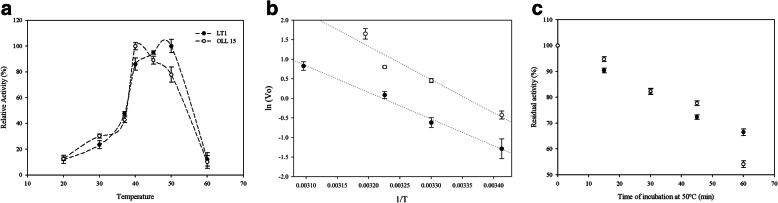
**a** Effect of the temperature on the activity of the keratinolytic extracts obtained with *Bacillus cytotoxicus* LT-1 and *Bacillus cytotoxicus* Oll-15. **b** Arrhenius plot obtained by plotting the logarithm of enzymatic activity (Vo) versus inverse of temperature (1/T, in Kelvins); LT-1 (black) and Oll-15 (white). **c** Effect of temperature on enzyme stability**.** Residual activity in function of time incubation at 50 °C. Each determination was performed in triplicates, and values represent mean ± SD

Regarding the optimum temperature for proteolytic activity, it could be seen that *Bacillus cytotoxicus*’ enzymatic extracts differ from each other. *B. cytotoxicus* LT-1 proteolytic extract displayed an optimum temperature of 50 °C, while *B. cytotoxicus* Oll-15 displayed an optimum temperature of 40 °C. At 60 °C, both extracts presented a considerably low relative activity (12% and 10% for LT-1 and Oll-15, respectively), and no activity was detected above 60 °C (Fig. 3a).

Activation energies for the reaction were calculated using the Arrhenius plot and resulted in 56.66 KJ/mol for LT-1 enzyme cocktail and 70.69 KJ/mol for Oll-15 enzyme cocktail, respectively (Fig. 3b).

Thermostability study showed that *Bacillus cytotoxicus* LT-1 and Oll-15 proteolytic extracts were completely stable up to 45 °C and retained approximately 66.5% and 71.3% of their activity at 50 °C after 60 min of incubation (Fig. 3c). The half-life times of LT-1 and Oll-15 extracts were 99 and 115.5 min, respectively at 50 °C. It was observed that at 60 °C, both extracts were rapidly denatured, and no activity was detected after 15 min of incubation.

### Effect of chemicals on proteolytic activity

Proteolytic activity of *B. cytotoxicus* extracts were completely inhibited by EDTA and 1,10 phenanthroline indicating that proteolytic enzymes present in these extracts belong to the group of metalloproteases (Table 1). Regarding the effect of certain organic solvents, they slightly inhibited the activity of the extracts. The presence of ethanol reduced the LT-1 activity by 13% and did not affect Oll-15 activity. Isopropanol produced an inhibition between 13 and 15%. Methanol did not affect the activity in neither of the extracts.

**Table 1:** Effect of protease inhibitors, organic solvents, metal ions, and detergents on the activity of the keratinolytic extracts obtained with *Bacillus cytotoxicus* LT-1 and *Bacillus cytotoxicus* Oll-15. Each determination was performed in triplicate. Residual activity is expressed as a percentage, comparing with the activity measured in the absence of any compound. Asterisks show the differences with a *P* < 0.05 (LSD test, ANOVA).

Chemical	Concentration	Residual activity (%)	
		LT-1	Oll-15
Inhibitors			
PMSF	1 mM	87.8 ± 3.0*	95.8 ± 4.4*
Iodoacetamide	1 mM	89.7 ± 4.2*	56.9 ± 3.2*
EDTA	1 mM	4.5 ± 1.9*	0.6 ± 0.1*
Phenantroline	1 mM	1.8 ± 0.5*	0.8 ± 0.5*
Pepstatin A	5 μM	98.5 ± 1.2	111.1 ± 0.4*
Metal ions			
Mg^+2^	1 mM	121.8 ± 2.2*	130.1 ± 2.9*
Zn^+2^	1 mM	107.5 ± 5.3*	112.2 ± 10.0*
Ca^+2^	1 mM	143.8 ± 2.3*	147.2 ± 0.8*
Mn^+2^	1 mM	117.5 ± 3.1*	116.2 ± 13.8*
Co^+2^	1 mM	41.3 ± 4.4*	46.4 ± 3.3*
Na^+^	1 mM	94.9 ± 6.7	82.7 ± 6.5*
K^+^	1 mM	89.1 ± 4.2*	107.1 ± 6.4
Hg^+2^	1 mM	28.5 ± 3.1*	27.9 ± 2.3*
Organic solvents			
Methanol	1% v/v	98.3 ± 1.5	102.6 ± 1.7
Ethanol	1% v/v	87.4 ± 3.7*	97.8 ± 2.7
Isopropanol	1% v/v	84.9 ± 0.7*	87.3 ± 1.2*
Commercial detergents			
Ala	1% v/v	45.3 ± 3.2*	9.0 ± 1.1*
Ariel	1% v/v	15.9 ± 1.4*	17.8 ± 6.6*
Ace	1% v/v^-^	42.3 ± 2.1*	1.9 ± 1.5*
Skip	1% v/v	20.1 ± 1.1*	1.2 ± 2.1*
Drive	7 mg·l^-1^	9.3 ± 1.5*	5.0 ± 1.5*
Ariel	7 mg·l^-1^	4.1 ± 1.2*	2.0 ± 0.7*
Ace	7 mg·l^-1^	10.7 ± 1.4*	39.1 ± 1.5*
Skip	7 mg·l^-1^	4.7 ± 1.3*	2.1 ± 0.6*

The effect of different metal ions is shown in Table 1. Divalent cations Ca^+2^, Mg^+2^, Mn^+2^, and Zn^+2^ increased protease activity by 143.8, 121.8, 117.5, and 107.5% for LT-1, respectively, and by 147.2, 130.1, 116.2, and 112.2% for Oll-15, respectively.

### SDS-PAGE and zymogram

**Fig. 4:**
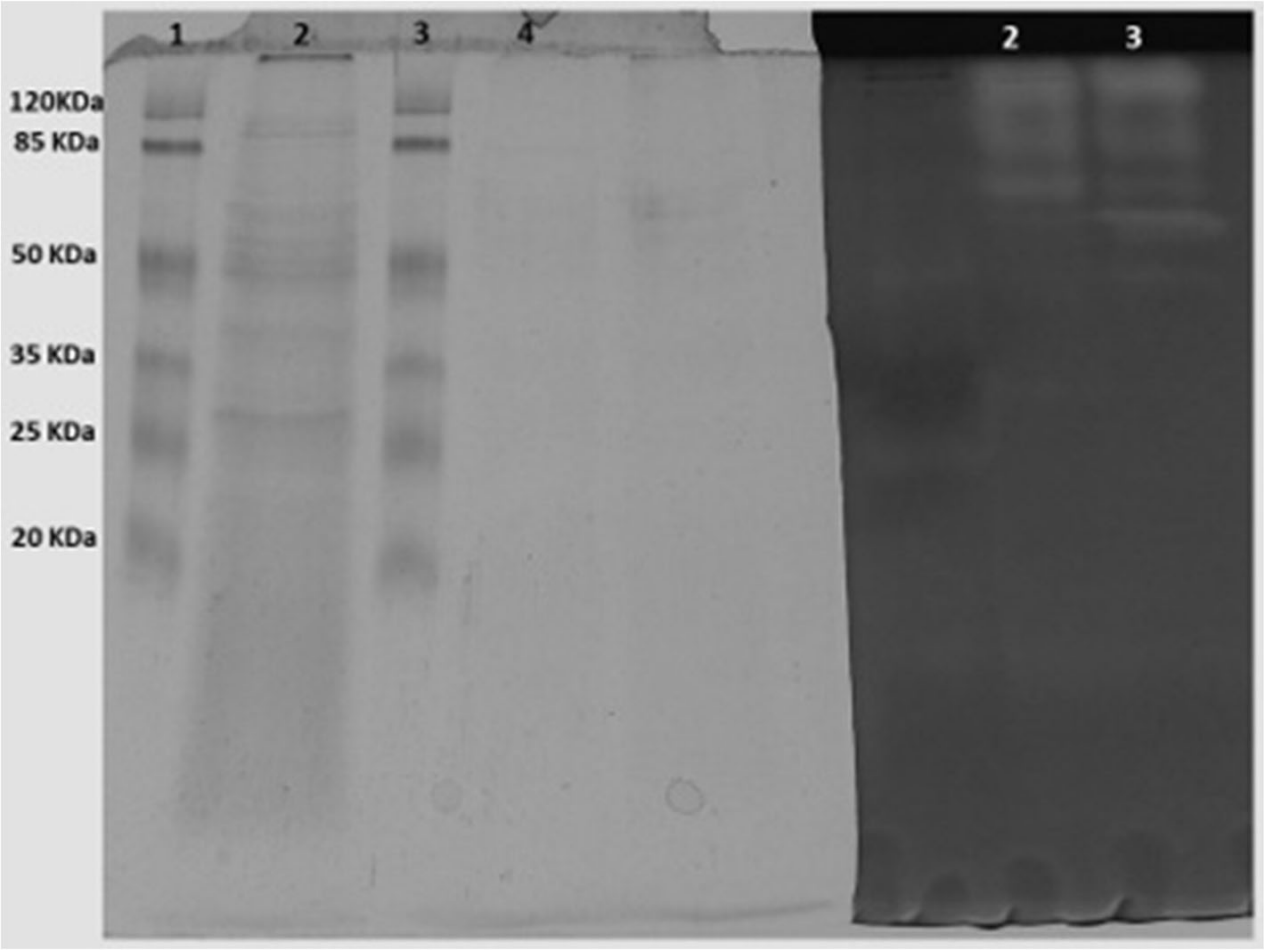
SDS-PAGE (left) and Zymogram (right) of the keratinolytic extracts. From left to right: molecular weight marker (lane 1), LT-1 crude extract (lane 2), molecular weight marker (lane 3), Oll-15 crude extract (lane 4). Zymogram: LT-1 crude extract (lane 1) and Oll-15 crude extract (lane 2)

An SDS-PAGE coupled to a casein zymogram was performed in order to estimate the molecular weight of the proteases present in the extracts (Fig. 4). At least three different proteolytic bands were visualized on the LT-1 zymogram (Lane 2), two of them with apparent molecular weights between 50 and 85 kDa and one band with a molecular weight higher than 120 kDa. The zymogram of the crude extract of Oll-15 showed a similar proteolytic composition, but with an additional band slightly over 50 kDa (Lane 3).

### Stability and compatibility with commercial laundry detergents

The data presented in Table 1 show that although both enzymatic extracts lost a considerable amount of activity when they were incubated with commercial detergents, it is worthy to mention that *B. cytotoxicus* LT-1 proteases retained 45.3% residual activity after 1 h of incubation in Ala and 42.3% in Ace, both liquid detergents, while the maximum stability for *B. cytotoxicus* Oll-15 was observed with the solid detergent Ace (39.1%).

### Bioactivities of FPHs: characterizations of the feather protein hydrolysates

We explore the antioxidant activity of the FPHs obtained by the action of the proteases of *B. cytotoxicus* LT-1 and Oll-15 by two in vitro methods: the reducing power of Fe^+3^ and the radical scavenging activity assays (ABTS assay). In general, dose-dependent responses were observed, and an increase of FPH concentrations resulted in enhanced bioactive potentials.

**Fig. 5:**
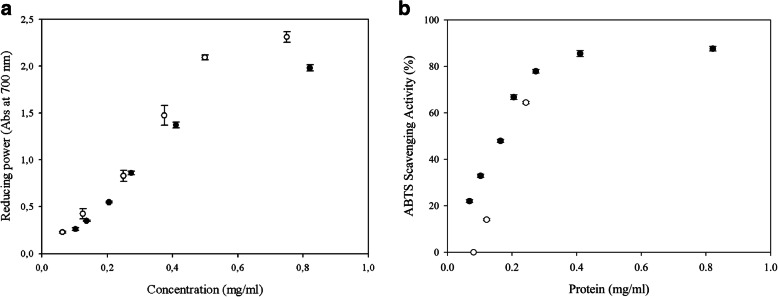
**a** Reducing power of the feather protein hydrolysate produced by *Bacillus cytotoxicus* Oll-15 (black) and ascorbic acid (white). **b** ABTS scavenging activity (%) of the feather protein hydrolysates produced by *Bacillus cytotoxicus* LT-1 (white) and *Bacillus cytotoxicus* Oll-15 (black)

The FPH obtained from *B. cytotoxicus* LT-1 biodegradation did not show an appreciable activity of reducing power, while the FPH from *B. cytotoxicus* Oll-15 biodegradation (protein concentration: 0.27 mg·ml^-1^) produced an absorbance (700 nm) of 0.86, similar to that obtained by a solution of 0.25 mg·ml^-1^ of ascorbic acid (Abs_700_ 0.83, Fig. 5a).

Regarding ABTS radical scavenging activity, both LT-1 and Oll-15 FPHs demonstrated a considerable antioxidant activity: 10.17 and 11.74 mEq of Trolox/g of protein, respectively. In terms of scavenging activity, the activity obtained by the extracts was 85% and 64% (0.41 mg·ml^-1^ of protein from Oll-15 FPH and 0.24 mg·ml^-1^ of protein from LT-1 FPH, respectively, Fig. 5b).

The EC_50_ values were calculated from the dose–response curves being 0.16 mg·ml^-1^ for FPH obtained with *B. cytotoxicus* Oll-15 and 0.22 mg·ml^-1^ for FPH obtained with *B. cytotoxicus* LT-1.

## Discussion

As a proteolytic/keratinolytic enzyme producer, *Bacillus* is one of the most studied genera. Their keratinolytic proteases have been studied since 1990 when *Bacillus licheniformis* PWD-1—a feather-degrading bacterium isolated from a thermophilic poultry waste digestor—was reported [24]. At 50 °C, a complete degradation of keratin protein by *B. licheniformis* PWD-1 was observed after 7 to 10 days [24]. Although keratinolytic proteases have been studied since 90s, keratinases from thermophilic or thermotolerant *Bacillus* is not common, being the list of this type of keratinolytic microorganisms quite short. *B. licheniformis* K-508 [25], *B. subtilis* RM-01 [26], *Brevibacillus thermoruber* T1E [27], and *B. halodurans* JB99 [28] are representatives of this group of microorganisms.

Our work has the aim to enlarge the list of these moderate extremophiles studying the production and characterization of the proteolytic enzymes produce by novel strains of the thermotolerant keratinolytic bacteria: *Bacillus cytotoxicus*. *Bacillus cytotoxicus* strains have been isolated for the first time from pristine areas in 2020 [11].

Under submerged fermentation with feathers as only source of carbon and nitrogen, the maximum enzyme activity was reached after 24–33 h for *B. cytotoxicus* Oll-15 and LT-1, respectively at 50 °C. This agrees with Bouacem et al. [29] who reported the thermophilic bacteria *Caldicoprobacter algeriensis* isolated from hot spring, which produced 21.0 U/ml at 50 °C after 24 h of incubation. The mesophilic *B. cereus* LAU08 and TS1 had an optimum incubation time of 48 and 72 h, respectively [30, 31]. Meanwhile, enzyme productions by *B. pumilus* A1 [32] and *B. weihenstephaensis* PKD5 [33] had optimum incubation periods of 4 and 7 days, respectively.

Regarding the biochemical characterization it could be said that, in general, *Bacillus*` proteases—with keratinolytic activity—showed basic optimum pHs in the range of 8.0–9.0 (i.e., *B. subtilis*, *B. licheniformis* ALW1, and *B. pumilus* FH9) [34–37]. *B. amyloliquefaciens* S13 and *Bacillus* sp. P45 keratinolytic crude proteases are examples of those that showed an optimum pH in the neutral range (6.5–7.0) [36]. Higher optimum pH values were generally observed on thermophilic and thermotolerant proteases. *Thermoanaerobacter* sp. 1004-09, *Streptomyces* sp. AB1, and *Actinomadura keratinolytica* Cpt29 keratinases have alkaline optimum pHs in the range of 9.0 to 11.5 [7, 38, 39]. *Clostridium* PE’s keratinase also showed an optimum pH in a neutral to alkaline range, between 7.0 and 9.0 [8].

*Bacillus cytotoxicus* crude extracts present optimum temperatures that are in concordance with other proteases with keratinolytic activity from the genera *Bacillus*. Usually, these enzymes displayed optimum temperatures in the range of 50 to 60 °C. *B. licheniformis* ALW1 produced a keratinase which was reported to have an optimum temperature of reaction of 65 °C [40]. Cai et al. [34] purified a keratinase produced by *B. subtilis* KD-N2, with an optimum temperature of 55 °C. Hamiche et al. [36] and Abdel-Naby et al. [35] studied keratinases from *B. amyloliquefaciens* S13 and *B. pumilus* FH9, reporting its optimum temperatures between 50 and 60 °C. *Bacillus* sp. P45 crude keratinolytic protease displayed optimum activity at 50 °C [41]. *Bacillus aerius* NSMk2 keratinase showed optimum activity at 45 °C [42], while the optimum temperature for *Bacillus subtilis* NRC3 keratinase was 40 °C [43].

Activation energies calculated for both extracts are in concordance with their thermotolerant characteristic, being higher than other results obtained from non-thermotolerant proteases. Higher free energies of activation and decrease in the unfolding rate are cited as mechanisms of adaptation, mainly on thermophilic and hyperthermophilic proteins [44]. Abdel-Naby et al. [35] and Hashem et al. [45] reported activation energies of 24 KJ/mol and 25.3 KJ/mol for the proteases with keratinolytic activity from the mesophylls *B. pumilus* FH9 and *B. licheniformis* ALW1.

Thermal stability of proteolytic extracts of *B. cytotoxicus* strains was a common feature that it is observed among *Bacillus* sp. Proteases and keratinases. *B. invictae* enzymatic preparation retained 94% of its original activity after 60 min at 50 °C [46]. In the case of *B. subtilis* FTC02PR1 keratinolytic enzymes showed a rapid activity loss after incubation for 15 min at 55 °C [47]. In addition, Gegeckas et al. [48] reported that the half-life time of BPKer from *Bacillus* sp. AD-W was 44 min at 50 °C, while the keratinase produced by *B. licheniformis* ALW1 had a half-life of 2 h at 60 °C [40].

The sensitivity of the enzymes under investigation to the protease inhibitors, EDTA and 1,10 phenanthroline, suggests that they belong to the metallo-class of keratinase. Also, the inhibition of Oll-15 extract by Iodoacetamide suggests the presence of free cysteine near the active site of the enzymes [49]. Although most of the keratinases investigated until now can be classified as serine proteases, keratinolytic metalloproteases from the *Bacillus* spp. have gained prominence. Metalloproteases are from *Bacillus* sp. P45, *Bacillus* sp. SCB-3, *Bacillus subtilis* MTCC (9102), *Bacillus polymyxa* and *B. cereus*, *Bacillus* sp. CSK2, etc. [41, 50–53].

The inhibitory effect of organic solvents on proteases from the *Bacillus* species has been reported earlier. Methanol, ethanol, and isopropanol inhibited the activity of the protease from *Bacillus subtilis* KD-N2 by 21%, 38%, and 24%, respectively, while activity of BPKer from *Bacillus* sp. AD-W was inhibited by 22.4%, 44% and 41.2%, respectively [34, 48]. A stronger inhibition was observed when the alkaline protease BS1 from *B. safensis* S406 was incubated with methanol and ethanol (10.2% and 7.1% of residual activity, respectively) [54]. Generally, hydrophilic solvents with log *P* values below 4.0 are considered extremely toxic e-methanol (log *P* = − 0.76, ethanol (log *P* = − 0.24) and isopropanol (log *P* = 0.28) [54, 55]. Although these solvents are classified as extremely toxic, the keratinolytic enzymes produce by *B. cytotoxicus* strains were slightly inhibited by them.

Metallo-keratinases usually displayed variable metallic ion tolerance. It has been reported that divalent cations help the maintenance of the active site conformation and are important to stabilize the enzyme–substrate complex [56]. Co^+2^ and Hg^+2^ inhibited the enzymatic activity on both extracts, with 28–46% of residual activity (Table 1). The inhibition by heavy metals is commonly observed on hydrolytic enzymes, and the inhibition by Hg^+2^ may suggest the presence of a free cysteine near the active sites [57].

Most proteases and keratinases produced by bacteria are monomers with molecular weight varying from 14 to 240 kDa [1]. Most common molecular weight from bacterial proteases with keratinolytic activity rounds near 30 kDa and higher molecular masses were related to metalloproteases from thermophilic microorganisms [56]. Riessen and Antranikian [6] report a keratinase of 135 kDa produced by *Thermoanaerobacter keratinophilus*, and Kublanov et al. [7] report a keratinase of 150 kDa produced by *Thermoanaerobacter* sp. 1004-09. A keratinase of 35 kDa produced by the thermophilic strain *Thermoactinomyces s*p. YT06 was recently reported by Wang et al. [58].

Keratinolytic proteases from *B. cytotoxicus* strains demonstrated similar residual activity after the incubation with selected commercial laundry detergents. Paul et al. [59] reported that *Paenibacillus woosongensis* crude keratinase showed a range of 48.1–70.4% residual activity under similar treatment conditions. Some ingredients of detergents including fiber brighteners, foam regulators, bleaching agents, re-deposition agents, surfactants, and softening builders have been reported to affect the stability of proteolytic enzymes [60].

It is well known that proteases and keratinases are required in food, biomedical, pharmaceutical, and cosmetic industries. Currently, proteolytic microorganisms with the ability to produce feather protein hydrolysates (FPH) have gained territory because its utilization involves the revalorization of a residue to obtain a by-product: an animal-food additive [56]. The degradation of the feather on a minimal media can improve the nutritional value of keratinous wastes [56].

The feature that non-reducing power was observed in FPH obtained from *B. cytotoxicus* LT-1 and that a good reducing power was observed in Oll-15 FPH demonstrated that the composition of proteolytic/keratinolytic cocktails is important when an FPH with bioactive compounds is looking for Oll-15 FPH with 0.27 mg·ml^-1^of soluble protein producing an absorbance (Abs 700 nm) of 0.86. This value is higher than that reported by Callegaro et al. [61] for an FPH produced by *Bacillus* sp. C18 during submerged cultivation after 24 h, where 0.15 mg·ml^-1^ of soluble protein produced 0.068 Abs 700 nm; moreover, 3.68 mg·ml^-1^ of soluble protein (after 7 days of cultivation) produced just 0.318 Abs 700 nm. Similar results were obtained for an FPH produced by *Bacillus* sp. CL14; after 13 days of cultivation, 3.51 mg·ml^-1^ of soluble protein produced 0.230 Abs 700 nm.

The scavenging activity of FPHs from *B. cytotoxicus* degradation is in concordance with other authors and is even higher. Fontoura et al. [62] reported that FPH (15–25 mg·ml^-1^ of protein) produced by *Chryseobacterium* sp. kr6 has 95% ABTS scavenging activity, while FPH with 3.51 mg·ml^-1^ of soluble protein present on *Bacillus* sp. CL14 FPH has 75.95% ABTS scavenging activity and FPH with 0.663 mg·ml^-1^ of soluble protein has 35.23% ABTS scavenging activity in the case of *Bacillus* sp. CL33A [61].

The EC_50_ values were calculated from the dose–response curves, 0.16 mg·ml^-1^ for FPH obtained with *B. cytotoxicus* Oll-15 and 0.22 mg·ml^-1^ for FPH obtained with B*. cytotoxicus* LT-1. Using ABTS assay, FPHs obtained with *Chryseobacterium* sp. Kr6 presented lower antioxidant activity (EC_50_ of 16.0–18.3 mg·ml^-1^, [62]) than the FPH obtained with *Bacillus* sp. CL18 and lower than those reported here.

Although further studies are required to elucidate the significance of the isolates in the application in biotechnology, this first approach demonstrates that thermotolerant microorganisms are a niche that is needed to be investigated in order to find new biocatalysts producing new robust enzymes and bioactive peptides.

## Conclusions

This work represents the first study related to the production and characterization of proteases produced by *Bacillus cytotoxicus* strains. Bacterial strains studied on this work represent a novel source of proteases with keratinolytic activity, which are of industrial interest. Enzymatic extracts demonstrated to be a cocktail of different proteolytic enzymes of metalloprotease nature that were biochemically characterized.

When the FPHs obtained were assessed for antioxidant activity, it was found that they possess good antioxidant activity demonstrating its potential application as an additive for animal feed formulations. However, this potential application requires further investigations of the safety of FPHs. It is worth mentioning that previous reports [10, 63] stand out in that the detection of *cytK-1* gene is not a sufficient criterion for identification as cytotoxic strains.

## Data Availability

All data generated or analyzed during this study are included in this published article.

## Abbreviations

FPH: Feather protein hydrolysate
EC50: Half maximal effective concentration
MMM: Minimal mineral media
TCA: Trichloroacetic acid
SDS: Sodium dodecyl sulfate
